# Efficacy and safety of Chinese patent medicine (Jinlong capsule) in the treatment of advanced hepatocellular carcinoma: a meta-analysis

**DOI:** 10.1042/BSR20194019

**Published:** 2020-01-17

**Authors:** He Xu, Wenjie Wei, Mu Y., Chengwei Dong

**Affiliations:** 1 Infection Control Office, Weifang People’s Hospital, Weifang 261041, Shandong Province, China; 2 Department of Infectious Diseases, Weifang People’s Hospital, Weifang 261041, Shandong Province, China; 3 Department of Gastroenterology, Liaocheng People’s Hospital, Liaocheng 252000, Shandong Province, China; 4 Department of Hepatobiliary Surgery, Weifang People’s Hospital, Weifang 261041, Shandong Province, China

**Keywords:** conventional treatment, hepatocellular carcinoma, Jinlong Capsule, meta-analysis, traditional Chinese medicine

## Abstract

Jinlong capsule (JLC), a type of herbal medicine, is considered to be a promising adjuvant therapy for hepatocellular carcinoma (HC). Although an analysis of the published literature has been performed, the exact effects and safety of JLC are yet to be systematically investigated. Therefore, a wide-ranging systematic search of electronic databases to draw conclusions was performed. Data from 29 trials, including 2488 patients with advanced HC, were analyzed. The results indicated that, compared with conventional treatment alone, the combination of conventional treatment and JLC markedly improved overall patient response (odds ratio (OR) 2.06 [95% confidence interval (CI) 1.71–2.49]; *P*<0.00001), disease control rate (DCR) (OR 2.17 [95% CI 1.74–2.71]; *P*<0.00001) and quality of life (QoL) (OR 2.71 [95% CI 2.05–3.58]; *P*<0.00001), and significantly prolonged 6- (*P*=0.01), 12- (*P*<0.00001), 24- (*P*=0.001) and 36-month (*P*<0.0001) overall survival (OS) rates. The immune function of patients was also significantly enhanced after combined conventional therapy and JLC treatment, indicated by clearly increased percentages of CD3^+^ (*P*<0.0001), CD4^+^ (*P*<0.00001) and natural killer (NK) cells (*P*=0.0003), and CD4^+^/CD8^+^ ratio (*P*<0.00001). The incidence of leukopenia (*P*<0.00001), hepatotoxicity (*P*=0.005), and myelosuppression (*P*=0.0007) was lower in HC patients injected with JLC, whereas other adverse events did not differ significantly between the two groups (*P*>0.05). In summary, results of this meta-analysis suggest that the combination of conventional treatment and JLC is more effective for the treatment of HC than conventional treatment alone.

## Introduction

Hepatocellular carcinoma (HC) is the seventh most common cancer and the third leading cause of cancer-related deaths worldwide [1]. It was estimated that 840000 new cases of HC and 781631 HC-related deaths occurred worldwide in 2018 [1]. Despite advances in diagnostic methods, early detection of HC remains difficult [2]. In most patients, HC progresses to the intermediate and advanced stages, for which the 5-year survival rate is <17% [2,3]. Surgery and liver transplantation are considered to be the optimal treatment options for advanced HC; however, only a small proportion of HC patients are able to undergo potentially curative resection [4]. In addition, the therapeutic effects of current conventional treatments for advanced HC, such as radiotherapy and chemotherapy, remain unsatisfactory [2,5,6]. Therefore, more effective, comprehensive treatment strategies are required.

Jinlong capsule (JLC) is a traditional Chinese medicine with the function of tonifying qi and blood, dredging collaterals and detoxification, and has been widely used as an effective adjuvant drug in cancer treatment [7,8]. It is traditionally prepared from three animals with medicinal properties: Bungarus, Agkistrodon and Gecko [7,8]. The active ingredients of JLC comprise at least 17 amino acids, including histidine, serine, arginine, glycine and aspartic acid, among others, which are extracted using modern cryogenic and biochemical separation techniques from freshly prepared animal drugs [8,9].

Research has shown that JLC can effectively reverse multiple-drug resistance in cancer cells and improve the efficacy of chemotherapy [10,11]. Many *in vitro* studies have demonstrated that JLC can suppress cellular mitotic division and inhibit proliferation principally by arresting the cell division cycle at S and G_2_/M [8,9]. It can also promote cancer cell apoptosis through activation of the pro-apoptotic proteins BNIP3, Bax and caspase-3, and down-regulating the expression of Bcl-2 for survival [8,9]. Several studies have indicated that conventional treatment combined with JLC is more effective for the treatment of advanced HC than conventional treatment alone [12,13]. Despite intensive studies, the clinical efficacy and safety of the combination of conventional treatment and JLC has not been systematically evaluated. In the present study, we conducted a meta-analysis to investigate the efficacy and safety of conventional treatment combined with JLC compared with conventional treatment alone for advanced HC, to provide a scientific reference for the design of future clinical trials.

## Materials and methods

The present meta-analysis was performed in accordance with the Preferred Reporting Items for Systematic Reviews and Meta-Analyses (PRISMA) guidelines, and the Cochrane Handbook. Ethics approval was not necessary due to the nature of the study (i.e. meta-analysis).

### Search strategy and selection criteria

A literature search was conducted using eight electronic databases, including the Web of Science, PubMed, Cochrane Library, Embase, Chinese Biological Medicine Database (CBM), China National Knowledge Infrastructure (CNKI), Chinese Scientific Journal Database (VIP) and the Wanfang database, for original articles published before February 2019. The search terms included: ‘Jinlong capsule’ combined with ‘liver carcinoma’ or ‘hepatocellular carcinoma’ or ‘liver cancer’ or ‘hepatocellular cancer’.

Studies fulfilling the following criteria were included in the meta-analysis: controlled trials investigating patients with advanced HC; those with >30 HC patients; studies comparing the clinical outcomes of conventional treatment plus JLC adjuvant therapy (experimental group) with conventional treatment alone (control group); and conventional treatments including transcatheter arterial chemoembolization, chemotherapy, radiotherapy, radiofrequency ablation, percutaneous ethanol injection, support and symptomatic treatment and ultrasound therapy.

Studies involving patients with mixed malignancies, non-controlled trials, non-clinical studies, literature reviews, meta-analyses, meeting abstracts, case reports, duplicate studies, experimental models and those with insufficient available data were excluded.

### Data extraction and quality assessment

Data were independently extracted by two investigators (H.X. and W.W.) according to the same inclusion criteria; disagreements were adjudicated by a third reviewer (C.D.). The extracted data were as follows: name of the first author; year of publication; tumor stage or Karnofsky Performance score (KPS); number of cases; intervention methods; control group regimens; dose of JLC and study parameters. To ensure the quality of the meta-analysis, the quality of the included randomized and non-randomized controlled trials was evaluated according to the Cochrane Handbook tool [14] and Methodological Index for Nonrandomized Studies (MINORS, Supplementary Table S2), respectively [15].

### Outcome definitions

Clinical responses included treatment efficacy, quality of life (QoL), immune function, and adverse events. Treatment efficacy was assessed in terms of overall survival (OS), overall response rate (ORR), and disease control rate (DCR). Patient QoL was evaluated using QoL improved rate (QIR) and KPS. Immune function indicators (CD3^+^, CD4^+^, CD8^+^ and natural killer [NK] cell percentage and CD4^+^/CD8^+^ ratio) and adverse events, including leukopenia, adverse gastrointestinal effects, nausea and vomiting, anorexia, thrombocytopenia, hepatotoxicity, myelosuppression and anemia, were assessed and compared between the two groups.

### Statistical analysis

Review Mananger (RevMan) version 5.3 (Nordic Cochran Centre, Copenhagen, Denmark) and Stata version 13.0 (StataCorp., College Station, TX, U.S.A.) were used for statistical analyses. Data were mainly expressed as odds ratio (OR) with corresponding 95% confidence interval (CI), and a two-tailed *P*<0.05 was considered to be statistically significant. Cochrane’s Q test and *I^2^* statistics were used to assess heterogeneity among the studies: if *P*> 0.1 or *I^2^*  <  50%, fixed-effects model was used for the meta-analysis; otherwise, random-effects model was used [16].

The presence of publication bias was investigated using the Begg’s and Egger’s tests, and funnel plots. A pooled analysis of publication bias determined that the trim-and-fill method should be applied to coordinate the estimates from unpublished studies, and the adjusted results were compared with the original pooled OR [17]. Sensitivity analysis was performed to evaluate the impact of different therapeutic regimens, sample size, and type of research on the clinical efficacy of the combination of conventional treatment and JLC.

## Results

### Search results

**Figure 1:**
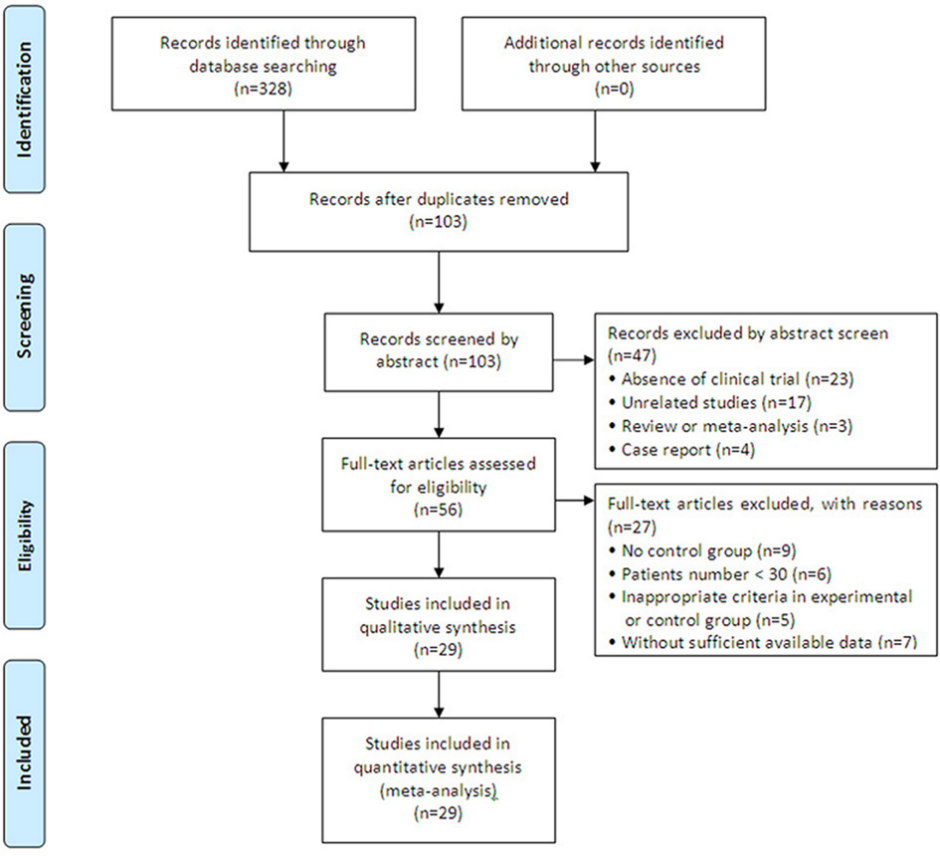
Study selection process for the meta-analysis

The initial search retrieved a total of 328 articles, of which 225 were excluded due to duplication. After title and abstract review, 47 articles were further excluded because they did not include clinical trials (*n*=23), were unrelated studies (*n*=17), were reviews and meta-analyses (*n*=3), or were case reports (*n*=4), leaving 56 studies as potentially eligible. After detailed assessment of full texts, studies without a control group (*n*=9), those with <30 HC patients (*n*=6), inappropriate criteria for the experimental or control groups (*n*=5), and trials with insufficient data (*n*=7) were excluded. Ultimately, 29 trials [7,18–45], involving 2488 patients with advanced HC, were included in the final analysis (Figure 1).

### Patient characteristics

All included studies were performed in different medical centers in China. In total, 1274 patients with advanced HC were treated using conventional methods in combination with JLC adjuvant therapy, while 1214 patients were treated using conventional methods alone. JLC was manufactured by Beijing Jiansheng Pharmaceutical Co., Ltd. The quality standards of the JLC in the present study were approved by the Chinese State Food and Drug Administration (SFDA), and granted a manufacturing approval number issued by the Chinese SFDA (Z10980041). All pharmaceutical companies involved followed the quality processing procedure outlined in the pharmacopeia. Study and patient characteristics are summarized in Table 1.

**Table 1:** Clinical information from the eligible trials in the meta-analysis

Included studies	Tumor stage/KPS	Patients Con/Exp	Intervening methods	Control group regimens	Dosage of JLC	Parameter types
Dong, H.T. (2008) [18]	II–IV	66/67	Con+JLC vs Con	TACE (THP, 5-Fu)	1.0 g/time, 3 times/day	OS, ORR, AE, QoL
Jia, C.H. (2008) [19]	I–III	30/30	Con+JLC vs Con	TACE (MMC)	1.0 g/time, 3 times/day	ORR, DCR, IF
Jiang, C.Y. (2013) [20]	II–III	33/30	Con+JLC vs Con	TACE (THP, 5-Fu)	1.0 g/time, 3 times/day	ORR, DCR, AE
Li, B. (2013) [21]	ND	73/74	Con+JLC vs Con	TACE (E-ADM, 5-Fu, DDP, MMC, CL)	1.0 g/time, 3 times/day	ORR, DCR, QoL
Li, H. (2007) [22]	ND	30/55	Con+JLC vs Con	TACE, RFA; PEI	1.0g/time, 3 times/day	OS
Liang, T.J. (2005) [23]	ND	108/116	Con+JLC vs Con	TACE (E-ADM, MMC, Carboplatin)	1.0 g/time, 3 times/day	OS, ORR, DCR
Liu, Z.Y. (2015) [24]	>60	80/80	Con+JLC vs Con	TACE (THP, MMC, 5-Fu, HCPT)	1.0 g/time, 3 times/day	ORR, DCR, QoL
Meng, P. (2016) [25]	ND	58/58	Con+JLC vs Con	TACE (E-ADM, 5-Fu)	1.0 g/time, 3 times/day	ORR, DCR, IF
Sun, J.H. (2006) [26]	ND	64/64	Con+JLC vs Con	SST	1.0 g/time, 3 times/day	OS, ORR, DCR, AE, QoL,
Tang, W.H. (2015) [27]	ND	20/20	Con+JLC vs Con	SST	2.0 g/time, 1 time/day	ORR, DCR, QoL
Wang, H.B. (2013) [28]	ND	42/43	Con+JLC vs Con	3-DCRT	1.0 g/time, 3 times/day	OS, ORR, DCR, AE
Wang, X.H. (2009) [29]	I–III	30/30	Con+JLC vs Con	TACE (DDP, MMC, 5-Fu)	1.0 g/time, 3 times/day	ORR, DCR, QoL, AE
Wu, G.L. (2010) [7]	≥60	45/53	Con+JLC vs Con	TACE (Oxaliplatin, HCPT, THP)	1.0 g/time, 3 times/day	ORR, DCR, QoL
Xiao, Z.Y. (2009) [30]	≥60	26/26	Con+JLC vs Con	3-DCRT	1.0 g/time, 3 times/day	ORR, DCR, AE
Xie, B. (2008) [31]	I–II	61/61	Con+JLC vs Con	Surgery, CT (5-Fu)	1.0 g/time, 3 times/day	OS, QoL, IF
Xie, Y.F. (2003) [32]	I–III	31/31	Con+JLC vs Con	TACE (DDP, MMC, 5-Fu)	1.0 g/time, 3 times/day	ORR, DCR, QoL, AE
Xiong, T.Q. (2010) [33]	≥60	26/26	Con+JLC vs Con	SST, KAI, SFJ	1.0 g/time, 3 times/day	ORR, DCR, AE
Yang, P.Y. (2013) [34]	≥60	36/34	Con+JLC vs Con	TACE (E-ADM, DDP, 5-Fu)	1.0 g/time, 3 times/day	ORR, DCR, QoL
Ye, X. (2008) [35]	II–IV	40/54	Con+JLC vs Con	HIFU	1.0 g/time, 3 times/day	OS, ORR, DCR
Yin, L.J. (2008) [36]	≥60	48/48	Con+JLC vs Con	GKS	1.0 g/time, 3 times/day	ORR, DCR, AE
Yuan, T.W. (2013) [37]	60–80	30/30	Con+JLC vs Con	TACE (DDP, MMC, 5-Fu)	1.0 g/time, 3 times/day	IF
Zeng, B.Z. (2010) [38]	I–III	30/31	Con+JLC vs Con	TACE (E-ADM, DDP, MMC)	1.0 g/time, 3 times/day	ORR, DCR, AE, IF
Zeng, C.S. (2012) [39]	II–III	30/30	Con+JLC vs Con	TACE (THP, DDP, 5-Fu)	1.0 g/time, 3 times/day	ORR, DCR
Zeng, C.S. (2014) [40]	II–III	30/30	Con+JLC vs Con	TACE (THP, DDP, 5-Fu)	1.0 g/time, 3 times/day	AE, IF
Zhang, S.Z. (2012) [41]	≥50	22/22	Con+JLC vs Con	SST	1.0 g/time, 3 times/day	ORR, DCR, AE
Zhang, X.Q. (2012) [42]	III–IV	77/74	Con+JLC vs Con	TACE (DDP, 5-Fu)	1.0 g/time, 3 times/day	OS, ORR, DCR, QoL, AE
Zhang, X.Q. (2012) [43]	>70	13/14	Con+JLC vs Con	TACE (Oxaliplatin, Gemcitabine)	1.0 g/time, 3 times/day	ORR, DCR, QoL, AE, IF
Zheng, C. (2018) [44]	II–III	28/30	Con+JLC vs Con	TACE (DDP, MMC, 5-Fu)	1.0 g/time, 3 times/day	ORR, DCR, AE, IF
Zhu, X. (2003) [45]	30–60	25/25	Con+JLC vs Con	SST	1.0 g/time, 3 times/day	ORR, DCR, QoL

Con, Control group (Conventional treatment alone group); Exp, Experimental group (Conventional treatment and JLC combined group). Abbreviations: AE, adverse event; CL, calcium levofolinate; CT, chemotherapy; DDP, Cisplatin; E-ADM, epirubicin; Fu, Fluorouracil; GKS, γ knife surgery; HCPT, hydroxycamptothecin; HIFU, high-intensity focused ultrasound therapy; IF, immune function; KAI, Kang Ai injection; MMC, mitomycin C; ND, non-determined; PEI, percutaneous ethanol injection; RFA, radiofrequency ablation; SFJ, compound injection of sophora flavescens; SST, support and symptomatic treatment; TACE, transcatheter arterial chemoembolization; THP, pirarubicin; 3-DCRT, three-dimensional conformal radiotherapy.

### Quality assessment

Quality assessment of the risk of bias is shown in Supplementary Figure S1 and Table S1. The results revealed that the literature retrieved for the present study was of good quality.

### Therapeutic efficacy assessments ORR and DCR

**Figure 2:**
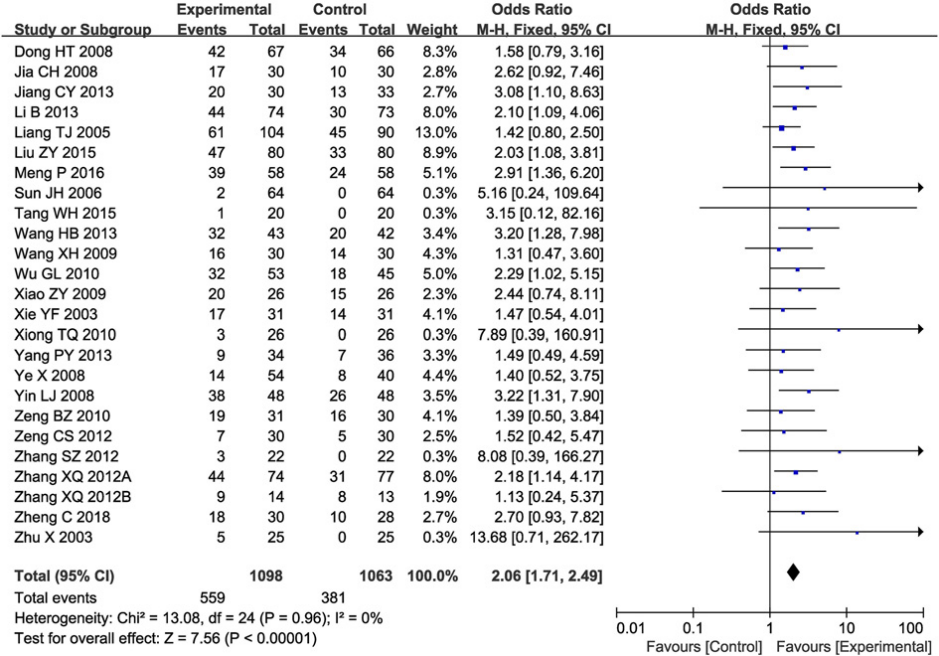
Forest plot of the comparison of ORR between the experimental and control groups Control group, conventional treatment alone group; Experimental group, conventional treatment and JLC combined group. The fixed-effects meta-analysis model (Mantel–Haenszel method) was used.

**Figure 3:**
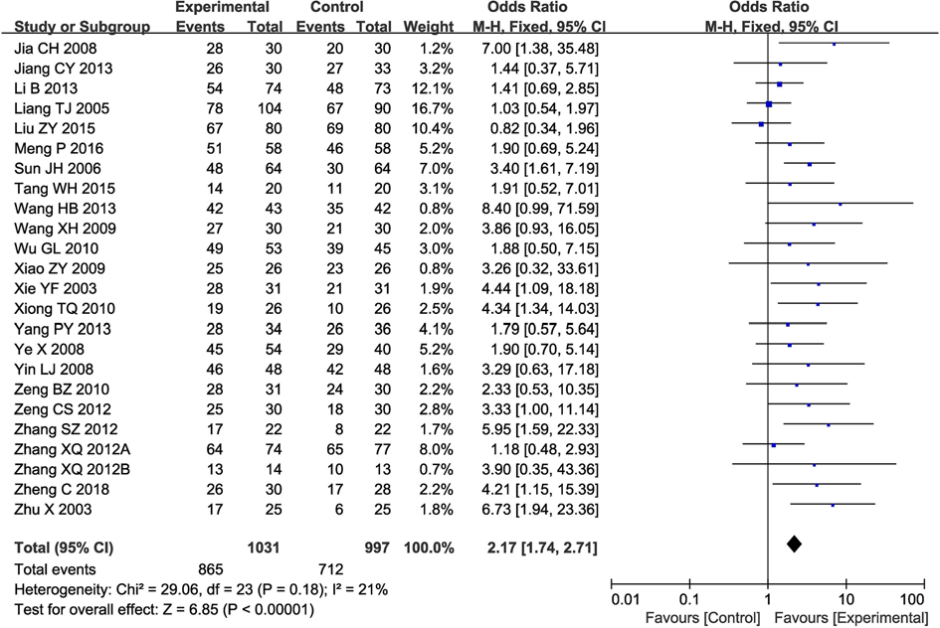
Forest plot of the comparison of DCR between the experimental and control groups Control group, conventional treatment alone group; Experimental group, conventional treatment and JLC combined group. The fixed-effects meta-analysis model (Mantel–Haenszel method) was used.

Twenty-five clinical trials [7,18–21,23–30,32–36,38,39,41–45], involving 2161 patients, compared ORR and DCR between the two groups. As shown in Figures 2 and 3, the pooled results revealed that patients who underwent combination therapy experienced significantly improved ORR (OR 2.06 [95% CI 1.71–2.49]; *P*<0.00001) and DCR (OR 2.17 [95% CI 1.74–2.71]; *P*<0.00001) compared with those who received conventional treatment(s) alone. The fixed-effect model was selected due to slight heterogeneity among the studies.

### Long-term survival

**Figure 4:**
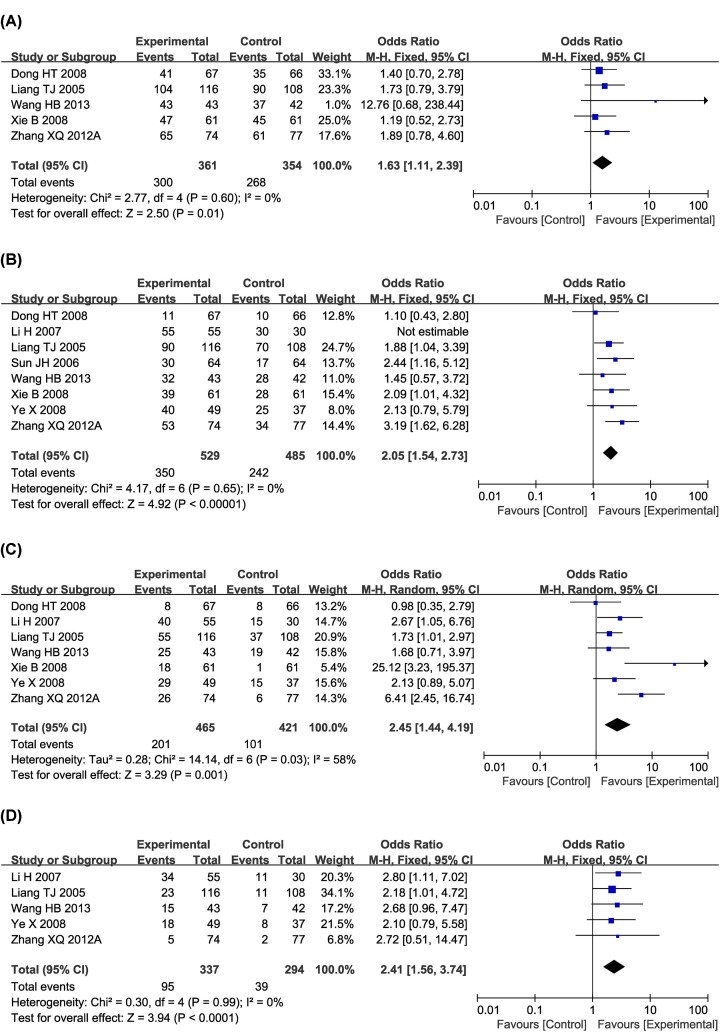
Comparisons of OS between experimental and control group Forest plot of the comparison of 6- (**A**), 12- (**B**), 24- (**C**) and 36-month (**D**) OS between the experimental and control groups. Control group, conventional treatment alone group; Experimental group, conventional treatment and JLC combined group.

Eight clinical trials [18,22,23,26,28,31,35,42] with 1014 HC patients reported OS (Figure 4). Meta-analysis revealed that the 6- (OR 1.63 [95% CI 1.11–2.39]; *P*=0.01), 12- (OR 2.05 [95% CI 1.54–2.73]; *P*<0.00001), 24- (OR 2.45 [95% CI 1.44–4.19]; *P*=0.001) and 36-month OS (OR 2.41 [95% CI 1.56–3.74]; *P*<0.0001) of patients in the combined treatment group were significantly prolonged compared with the control group. There was statistical heterogeneity in 24-month OS (*P*=0.03, *I^2^* = 58%) according to the heterogeneity test. Therefore, a random-effects model was used to pool this meta-analysis. Otherwise, the fixed-effect model was used.

### QoL assessment

**Figure 5:**
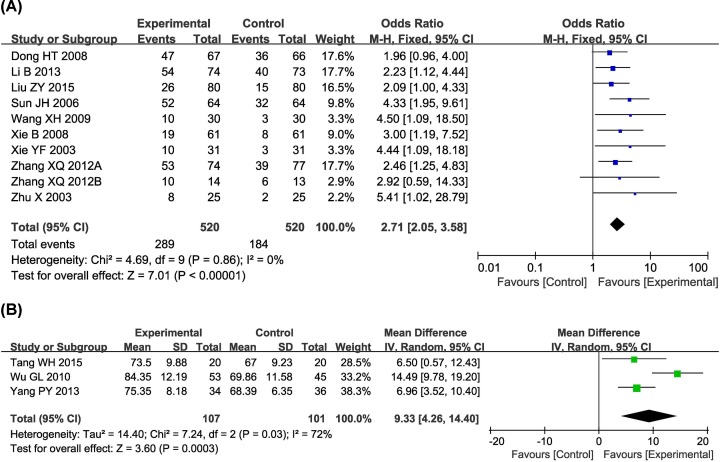
Comparisons of QoL between experimental and control group Forest plot of the comparison of QIR (**A**) and KPS (**B**) between the experimental and control groups. Control group, conventional treatment alone group; Experimental group, conventional treatment and JLC combined group.

Ten trials [18,21,24,26,29,31,32,42,43,45] with 1040 participants evaluated QIR, and three trials [7,27,34], including 208 patients, reported KPS data (Figure 5). Results demonstrated that the QoL of HC patients in the combined group was significantly better than that of the control group, indicated by significantly increased QIR (OR 2.71 [95% CI 2.05–3.58]; *P*<0.00001) and KPS (OR 9.33 [95% CI 4.26–14.40]; *P*=0.0003). QIR (*P*=0.86, *I^2^* = 0%) was not heterogeneous among the studies; therefore, a fixed-effect model was used to analyze OR. Otherwise, a random-effect model was used.

### Immune function evaluation

**Figure 6:**
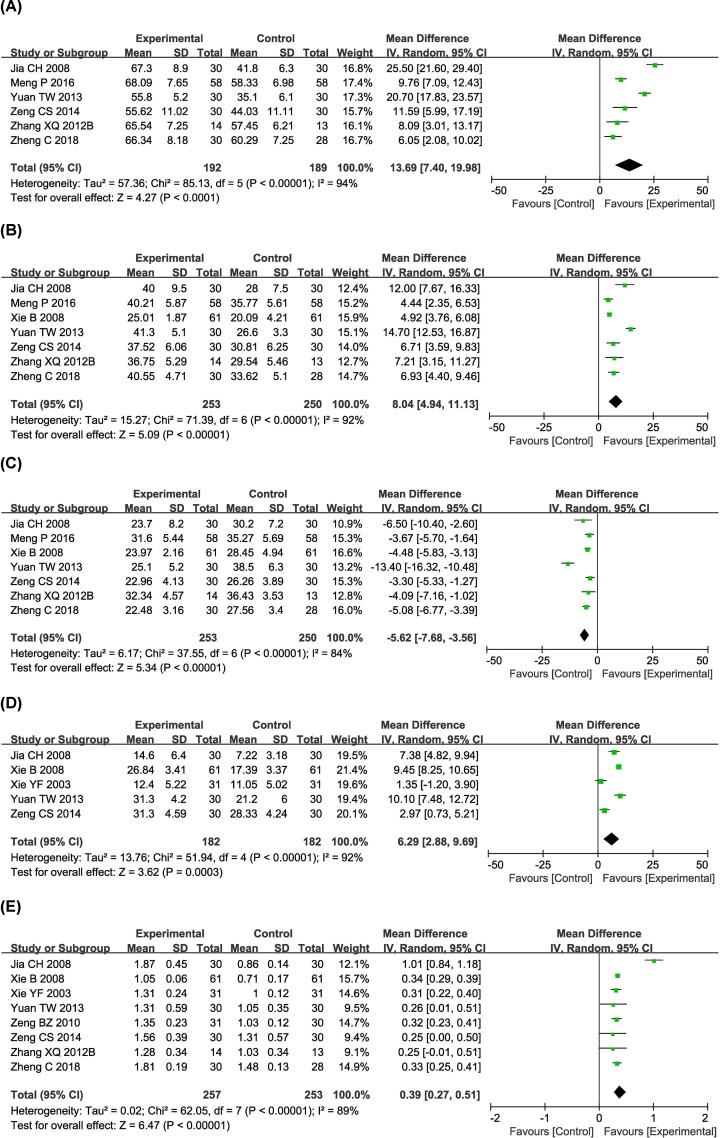
Comparisons of immune function between experimental and control group Forest plot of the comparison of immune function indicators including CD3^+^ (**A**), CD4^+^ (**B**), CD8^+^ (**C**) and NK (**D**) cells percentage and CD4^+^/CD8^+^ ratio (**E**) between the experimental and control groups. Control group, conventional treatment alone group; Experimental group, conventional treatment and JLC combined group.

Immune status of the patients was examined between the two groups in nine controlled studies [19,25,31,32,37,38,40,43,44]. The percentages of CD3^+^, CD4^+^ and NK cells, and CD4^+^/CD8^+^ ratio in the combined treatment group were significantly increased compared with those in the conventional treatment alone group, (CD3^+^, OR 13.69 [95% CI 7.40–19.98], *P*<0.0001; CD4^+^, OR 8.04 [95% CI 4.94–11.13], *P*<0.00001; NK, OR 6.29 [95% CI 2.88–9.69], *P*=0.0003; and CD4^+^/CD8^+^, OR 0.39 [95% CI 0.27–0.51], *P*<0.00001), whereas the proportions of CD8^+^ were clearly decreased (OR −5.62 [95% CI −7.68 to −3.56]; *P*<0.00001) (Figure 6). A random-effects model was used to pool this meta-analysis due to significant heterogeneity.

### Assessment of adverse events

As shown in Table 2 and Supplementary Figure S2, patients treated with JLC and conventional methods exhibited lower incidences of leukopenia, hepatotoxicity and myelosuppression (leukopenia, OR 0.49 [95% CI 0.36–0.67], *P*<0.00001; hepatotoxicity, OR 0.29 [95% CI 0.12–0.69, *P*=0.005; and myelosuppression, OR 0.26 [95% CI 0.12–0.56], *P*=0.0007), whereas analysis of gastrointestinal side effects, nausea and vomiting, anorexia, thrombocytopenia and anemia (gastrointestinal side effects, OR 0.69 [95% CI 0.44–1.07], *P*=0.10; nausea and vomiting, OR 0.81 [95% CI 0.45–1.48], *P*=0.49; anorexia, OR 0.89 [95% CI 0.35–2.29], *P*=0.81; thrombocytopenia, OR 0.76 [95% CI 0.28–2.06], *P*=0.59; and anemia, OR 0.68 [95% CI 0.29–1.60, *P*=0.38) did not differ significantly between the two groups. A fixed-effect model was used to analyze OR rate due to low heterogeneity.

**Table 2:** Comparison of adverse events between the experimental and control groups

Adverse events	Experimental group	Control group	Analysis method	Heterogeneity		OR	95% CI	*P*-value
	Number of patients (*n*) ref	Number of patients (*n*) ref		*I^2^* (%)	*P*-value			
Leukopenia	392	393	Fixed	8	0.37	0.49	0.36–0.67	<0.00001
Gastrointestinal adverse effects	195	195	Fixed	0	0.85	0.69	0.44–1.07	0.10
Nausea and vomiting	185	184	Fixed	0	1.00	0.81	0.45–1.48	0.49
Anorexia	112	112	Fixed	0	0.92	0.89	0.35–2.29	0.81
Thrombocytopenia	73	72	Fixed	0	0.84	0.76	0.28–2.06	0.59
Hepatotoxicity	60	61	Fixed	0	0.37	0.29	0.12–0.69	0.005
Myelosuppression	62	61	Fixed	0	1.00	0.26	0.12–0.56	0.0007
Anemia	73	72	Fixed	0	0.85	0.68	0.29–1.60	0.38

Control group, Conventional treatments alone group; Experimental group, Conventional treatments and JLC combined group.

### Publication bias

Publication bias was visually assessed using funnel plots (Supplementary Figure S3) and quantified using Egger’s test and Begg’s regression test (Supplementary Table S2). The funnel plots were symmetrical for 12- and 24-month OS, and CD4^+^, CD8^+^, CD4^+^/CD8^+^ and leukopenia, but were asymmetrical for ORR, DCR and QIR. To determine whether bias affected the pooled risk of ORR, DCR and QIR, a trim-and-fill analysis was performed. The adjusted OR indicated a trend similar to the results of the primary analysis (Supplementary Table S2), reflecting the reliability of the primary conclusions.

### Sensitivity analysis

A subgroup analysis was conducted to explore the source of heterogeneity for ORR and DCR. As shown in Supplementary Table S3, results revealed that no significant difference was found between the therapeutic regimens, sample size, and types of studies.

## Discussion

JLC, a type of traditional Chinese medicine, has been clinically applied as an adjuvant therapy for decades. Several studies have reported that addition of JLC could be beneficial to patients with advanced HC [12,13]. Although there was a statistical analysis of published clinical trials, the exact therapeutic effects are yet to be systematically evaluated because of small sample sizes and different protocols among various studies. Therefore, in the present analysis, we performed a wide-ranging online search according to strict inclusion and exclusion criteria, to draw a clear and systematic conclusion.

Data from 29 trials [7,18–45] including 2488 patients with advanced HC were included in our meta-analysis. JLC in all the included studies was manufactured by Beijing Jiansheng Pharmaceutical Co., Ltd. The dosages of JLC were 2.0–3.0 g per day via oral administration. The pooled results revealed that the combination of JLC and conventional treatment for HC achieved more beneficial effects compared with those treated solely with conventional therapy. Compared with conventional treatment alone, JLC could significantly improve ORR, DCR and QoL in patients with HC (*P*<0.05). The study also assessed whether JLC could prolong the long-term survival rates of HC patients, and the results showed that the 6-, 12-, 24- and 36-month OS of patients were significantly prolonged compared with the control group. These results indicated that using JLC could improve the short- and long-term curative effects of conventional treatment for advanced HC. However, the analysis of therapeutic effects may have been influenced by several factors. Therefore, we performed a subgroup analysis to determine the influence of different therapeutic regimens, sample sizes, study type and evaluation criteria on ORR and DCR. Subgroup analysis revealed that the therapeutic efficacy of JLC did not appear to be affected by these factors. However, there were limited studies and insufficient sample sizes for these investigations, which may have resulted in insufficient assessment. Therefore, these results need to be confirmed by new evidence.

The immunosuppressed status of cancer patients has been reported previously [2]. Therefore, immune system reconstruction is one of several critical aspects in effectively treating malignancies. Many studies have reported that JLC can enhance the ability of the body’s immunity and resistance to tumors [12]. Our analysis demonstrated that the percentages of CD3^+^, CD4^+^ and NK cells, and CD4^+^/CD8^+^ ratio, were significantly increased in HC patients treated with JLC, indicating that immune function in HC patients was improved after JLC adjuvant therapy.

Safety is the top priority of clinical treatment. Although JLC is cytotoxic to tumor cells, it may also be toxic to normal cells. As such, we analyzed 16 trials [18,20,26,28–30,32,33,36,38–44], involving 1192 patients, to evaluate adverse events according to World Health Organization standards. Meta-analysis revealed that patients who underwent JLC plus conventional treatment demonstrated a lower risk for leukopenia, hepatotoxicity and myelosuppression compared with conventional treatment alone, whereas analysis of other toxic side effects did not differ significantly. Therefore, JLC appears to be a safe auxiliary anti-tumor medicine for individuals with HC.

There were some limitations to our analysis. First, as an important Chinese patent medicine, JLC is mainly applied in China, which may lead to unavoidable regional bias. Second, several results demonstrated significant heterogeneity among the included trials, which may be due to the different tumor stages and ages of the HC patients and duration of treatment. However, based on the currently available literature, there are insufficient data to perform more statistical analysis to evaluate correlations. Third, our results may have inherent bias due to unclear randomization methods, allocation concealment and blinding in some of the included trials. Finally, there were limited studies and sample sizes for immune function and safety evaluation; therefore, analytical bias is possible. All these limitations may have resulted in insufficient evaluations of the outcome indicators.

## Conclusion

In summary, findings of this meta-analysis indicate that the combination of JLC and conventional treatment is effective in treating patients with advanced HC. The clinical application of JLC not only clearly enhanced the therapeutic effects of conventional treatment, but also effectively improved QoL and immune function in patients with HC. Thus, we anticipate that our study will provide valuable evidence for further evaluation of JLC. On the other hand, the low quality of some of the included publications increased the risk of bias, which, to some extent, affects the reliability of this research. Therefore, additional studies with high-quality evidence to verify the effectiveness of JLC-mediated therapy for HC are warranted.

## Supplementary Material

Supplementary Figures S1-S3 and Tables S1-S3

## Abbreviations

Bcl-2: B-cell lymphoma-2
BNIP3: B-cell lymphoma-2/adenovirus E1B nineteen kilodalton interacting protein-3
CI: confidence interval
DCR: disease control rate
HC: hepatocellular carcinoma
JLC: Jinlong Capsule
KPS: Karnofsky Performance Score
NK: natural killer
OR: odds ratio
ORR: overall response rate
OS: overall survival
QIR: quality of life improved rate
QoL: quality of life
SFDA: State Food and Drug Administration

## References

[1] Bray F., Ferlay J., Soerjomataram I., Siegel R.L., Torre L.A. and Jemal A. (2018) Global cancer statistics 2018: GLOBOCAN estimates of incidence and mortality worldwide for 36 cancers in 185 countries. CA Cancer J. Clin. 68, 394–424 10.3322/caac.21492 30207593

[2] Guo N., Miao Y. and Sun M. (2018) Transcatheter hepatic arterial chemoembolization plus cinobufotalin injection adjuvant therapy for advanced hepatocellular carcinoma: a meta-analysis of 27 trials involving 2,079 patients. Onco Targets Ther. 11, 8835–8853 10.2147/OTT.S182840 30573972 PMC6290874

[3] Zhu J., Yin T., Xu Y. and Lu X.J. (2019) Therapeutics for advanced hepatocellular carcinoma: recent advances, current dilemma, and future directions. J. Cell. Physiol. 234, 12122–12132 10.1002/jcp.28048 30644100 PMC13484161

[4] Filgueira N.A. (2019) Hepatocellular carcinoma recurrence after liver transplantation: risk factors, screening and clinical presentation. World J. Hepatol. 11, 261–272 10.4254/wjh.v11.i3.261 30967904 PMC6447422

[5] Hartke J., Johnson M. and Ghabril M. (2017) The diagnosis and treatment of hepatocellular carcinoma. Semin. Diagn. Pathol. 34, 153–159 10.1053/j.semdp.2016.12.011 28108047

[6] Lurje I., Czigany Z., Bednarsch J., Roderburg C., Isfort P., Neumann U.P. et al. (2019) Treatment strategies for hepatocellular carcinoma (-) a multidisciplinary approach. Int. J. Mol. Sci. 20, 10.3390/ijms20061465 30909504 PMC6470895

[7] Wu G.L., Zhang L., Li T.Y., Chen J., Yu G.Y. and Li J.P. (2010) Short-term effect of combined therapy with Jinlong Capsule and transcatheter arterial chemoembolization on patients with primary hepatic carcinoma and its influence on serum osteopontin expression. Chin. J. Integr. Med. 16, 109–113 10.1007/s11655-010-0109-9 20473734

[8] Li D., Ni T., Tao L., Jin F., Wang H., Feng J. et al. (2018) Jinlong Capsule (JLC) inhibits proliferation and induces apoptosis in human gastric cancer cells *in vivo* and *in vitro*. Biomed. Pharmacother. 107, 738–745 10.1016/j.biopha.2018.08.049 30138896

[9] Li Y., Hu J., Huang H. and He Y. (2013) Effect of Jinlong capsule on proliferation and apoptosis of human pancreatic cancer cells BxPC-3. J. Tradit. Chin. Med. 33, 205–210 10.1016/S0254-6272(13)60126-0 23789218

[10] Yang T.Y., Yi W., Wen J., Gan C.Y., Yang Y.C. and Dai M. (2019) Therapeutic effect of Jin Long capsule combined with neoadjuvant chemotherapy on invasive breast cancer and the expression change of multidrug resistance proteins. Chin. J. Oncol. 41, 118–123 10.3760/cma.j.issn.0253-3766.2019.02.008 30862141

[11] Lu Q., Luo J.B., Feng Y.F., She Q. and Shi Z.F. (2015) Jinlong capsule combined with chemoradiotherapy for NSCLC: a meta-analysis. China J. Chin. Mater. Med. 40, 4491–4496 27097429

[12] Zhang H.J., Yang J.J., Wang W.X., Jiang X., Mao Y.J., Yang C.A. et al. (2008) Effects of Jinlong Capsule on expressions of interleukin-2 and soluble interleukin-2 receptor in patients with primary liver cancer after transarterial chemoembolization therapy. J. Chin. Integr. Med. 6, 907–910 10.3736/jcim20080906 18782532

[13] Sun B.M., Wu M., Luo S.B. and Chen X.X. (2008) Jinlong Capsule combined with transarterial chemoembolization in treatment of gastric cancer with liver metastasis. J. Chin. Integr. Med. 6, 968–970 10.3736/jcim20080919 18782545

[14] Zeng X., Zhang Y., Kwong J.S., Zhang C., Li S., Sun F. et al. (2015) The methodological quality assessment tools for preclinical and clinical studies, systematic review and meta-analysis, and clinical practice guideline: a systematic review. J. Evid. Based Med. 8, 2–10 10.1111/jebm.12141 25594108

[15] Slim K., Nini E., Forestier D., Kwiatkowski F., Panis Y. and Chipponi J. (2003) Methodological index for non-randomized studies (minors): development and validation of a new instrument. ANZ J. Surg. 73, 712–716 10.1046/j.1445-2197.2003.02748.x 12956787

[16] Jackson D., White I.R. and Riley R.D. (2012) Quantifying the impact of between-study heterogeneity in multivariate meta-analyses. Stat. Med. 31, 3805–3820 10.1002/sim.5453 22763950 PMC3546377

[17] Lin L. and Chu H. (2018) Quantifying publication bias in meta-analysis. Biometrics 74, 785–794 10.1111/biom.12817 29141096 PMC5953768

[18] Dong H.T., Zhao W., Lu W.P., Chen L.Z., Yin Q.Z., Zhang Y. et al. (2008) Clinical observation on 133 cases of primary hepatocellular carcinoma treated by Jinlong Capsule and hepatic artery intervention therapy. Chin. J. Clin. Oncol. 35, 378–380

[19] Jia C.H., Wang W.Y. and Kang Y. (2008) Clinical studies on combination therapy of jinlong capsule and transarterial chemoembolization in treatment of primary hepatic carcinoma. Chin. J. Cancer Prev. Treat. 15, 1416–1418

[20] Jiang C.Y., Cao J.J. and Cheng Q. (2013) Clinical evaluation of Jinlong capsule combined with TACE in the treatment of primary hepatocellular carcinoma. Med. Innov. China 10, 110–111

[21] Li B., Zhao L.X., Liu Z.W., Li L., Ma L.B., Zhou Z.X. et al. (2013) Jinlong capsule combined with interventional therapy for primary hepatocellular carcinomas: a clinical analysis on 150 patients. Chin. J. Hepatobiliary Surg. 19, 530–533

[22] Li H., Zhang B. and Yu G.Y. (2007) Preventive effect of Jinlong Capsule on recurrence and metastasis of resectable hepatocellular carcinoma after operation. Capit. Med. 24, 35–36

[23] Liang T.J., Qin C.Y., Zhang C.Q. and Zhao X.Q. (2005) Clinical studies on the combination therapy with Jinlong Capsule and chemotherapy plus embolization by hepatic artery catheterization on primary hepatic carcinoma. Chin. J. Clin. Oncol. 32, 641–643

[24] Liu Z.Y., Zhang X.H., Zhang X.S., Li J.Y. and Ji X.W. (2015) Clinical observation of Jinlong Capsule combined with TACE in the treatment of primary hepatocellular carcinoma. Heilongjiang Med. Pharm. 38, 75–76

[25] Meng P. (2016) Clinical efficacy and mechanism of jinlong capsule plus hepatic artery embolism for primary liver cancer. Pract. J. Cancer 31, 403–406

[26] Sun J.H., Yang S.S. and Ma Y.L. (2006) Effect of Jinlong capsule on quality of life and survival period of advanced hepatocellular carcinoma patients. Hubei J. Trad. Chin. Med. 28, 34

[27] Tang W.H., She Z.C., Ding Z.X., Wang G.J. and Tang S.S. (2015) Effects of Jinlong capsule on expression of thioredoxin reductase in patients with advanced primary liver cancer. J. Hubei Univ. Chin. Med. 17, 35–36

[28] Wang H.B. and Yang J.Q. (2013) Combination therapy of Jinlong capsule and three-dimensional conformal radiotherapy for primary hepatocellular carcinoma. Chin. J. Clin. Oncol. 40, 784–787

[29] Wang X.H., Yang J.Q. and Li Y.H. (2009) Clinical observation of Jinlong capsule combined with interventional therapy for hepatocellular carcinoma. Chin. J. Integr. Trad. West Med. 29, 273–274

[30] Xiao Z.Y., Deng J.H., Xiong S.Z., Wang S. and Tang X.Y. (2009) Therapeutic effect of three-dimensional conformal radiotherapy combined with Jinlong Capsule on 52 cases of primary hepatocellular carcinoma. Capit. Med. 16, 45–46

[31] Xie B., Tang C. and Huang J. (2008) Effect of jinlong capsule combined with hepatectomy on HCC intrahepatic metastasis. Chin. J. Cancer Prev. Treat. 15, 1584–1586

[32] Xie Y.F., Tan X.F., Ma Q.H. and Liu S.Z. (2003) Clinical observation of Jinlong capsule combined with interventional therapy for hepatocellular carcinoma. Chin. J. Clin. Oncol. 30, 297–298

[33] Xiong T.Q., Wang Q.F. and Zhang Y. (2010) Analysis of Jinlong capsule for 26 cases of primary liver cancer patients. J. Med. Theory Pract. 23, 51–52

[34] Yang P.Y., Sun Y.Y., Zhang Y.C., Zhang X., Sun B.X. and Jia Y.J. (2013) Effectiveness of early intervention with Jin-long capsules and transarterial chemoembolization for the treatment of primary liver cancer. Chin. J. Clin. Oncol. 40, 45–49

[35] Ye X., Ge Z.M., Fei X.B., Wang S., Cheng Y.Y., Chen X.M. et al. (2008) Clinical study on HIFU combined with Jinlong capsules in treating 54 cases of primary liver cancer. Chin. J. Clin. Oncol. 35, 372–374

[36] Yin L.J., Zhao G.H., Ding T.G. and Peng Z.X. (2008) Jinlong capsule combined with whole body gamma knife in the treatment of 96 cases of primary liver cancer. Chin. J. Clin. Oncol. 35, 381–382

[37] Yuan T.W., Wen S.W., Dang Z.J., Zhang X.Q., Chang J.P. and Xue Y.Q. (2013) Regulation of immune functions by combined Jinlong capsule and interventional therapy in patients with primary liver cancer. Chin. J. Clin. Oncol. 40, 1116–1118

[38] Zeng B.Z., Yang Y.Q. and Han X.W. (2010) Jinlong capsule combined with TACE in the treatment of 31 cases of primary liver cancer. Trad. Chin. Med. Res. 23, 35–37

[39] Zeng C.S., Cai L.M., Li J.W., Huang Z.C., Xiao Y.H., Zhang C.Y. et al. (2012) Influence of Jinlong capsule combined with transarterial chemo-embolization on quality of life of patients with primary liver cancer. Chin. J. Clin. Oncol. 39, 1839–1842

[40] Zeng C.S., Cai L.M., Huang Z.C. and Xu Q.Y. (2014) The effect of Jinlong capsule on the immune function for intervened patients with primary liver cancer. Chin. German J. Clin. Oncol. 13, 80–83

[41] Zhang S.Z., Yang M.D. and Gao J. (2012) Clinical analysis of Jinlong capsule in the treatment of 22 cases of primary liver cancer. Capit. Med. 19, 35–36

[42] Zhang X.Q. (2012) Jinlong Capsule combined with chemotherapy for 14 cases of primary liver cancer with pulmonary metastasis. Jiangxi J. Trad. Chin. Med. 43, 35–37

[43] Zhang X.Q., Guo P., Dang Z.J. and Wen S.W. (2012) Jinlong Capsule combined with interventional therapy for hepatocellular carcinomas: clinical observation. J. Intervent. Radiol. 21, 249–251

[44] Zheng C., Zhang R.S., Pan Y. and Liu H.L. (2018) Effects of Jinlong capsule combined with interventional therapy on patients with primary hepatocellular carcinoma and its influence on t lymphocyte subsets and tumor immune factors. Modern Digest. Interv. 23, 506–509

[45] Zhu X. (2003) Clinical observation of jinlong capsule in the treatment of advanced primary hepatocellular carcinoma. Modern J. Integr. Trad. Chin. West. Med. 12, 1739–1740

