# Supplementary material for: Efficacy and safety of Chinese patent medicine (Jinlong capsule) in the treatment of advanced hepatocellular carcinoma: a meta-analysis
Source: Biosci Rep. 2020 Jan 17;40(1):BSR20194019. doi: 10.1042/BSR20194019 (PMC6970085; doi:10.1042/BSR20194019)
Supplement: Supplementary Figures S1-S3 and Tables S1-S3 [file BSR-2019-4019_supp.pdf]

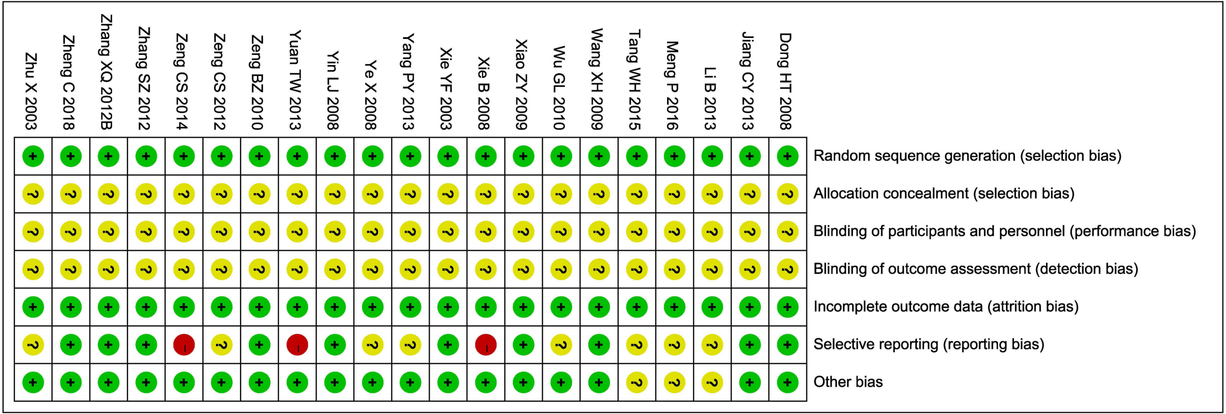
Zhu X 2003
Zheng C 2018
Zhang XQ 2012B
Zhang SZ 2012
Zeng CS 2014
Zeng CS 2012
Zeng BZ 2010
Yuan TW 2013
Yin LJ 2008
Ye X 2008
Yang PY 2013
Xie YF 2003
Xie B 2008
Xiao ZY 2009
Wu GL 2010
Wang XH 2009
Tang WH 2015
Meng P 2016
Li B 2013
Jiang CY 2013
Dong HT 2008
Random sequence generation (selection bias)
Allocation concealment (selection bias)
Blinding of participants and personnel (performance bias)
Blinding of outcome assessment (detection bias)
Incomplete outcome data (attrition bias)
Selective reporting (reporting bias)
Other bias

Supplementary Figure 1. Risk of bias summary: review of authors' judgments about each risk of bias item for included studies. **Note:** Each color represents a different level of bias: red for high-risk, green for low-risk, and yellow for unclear-risk of bias.

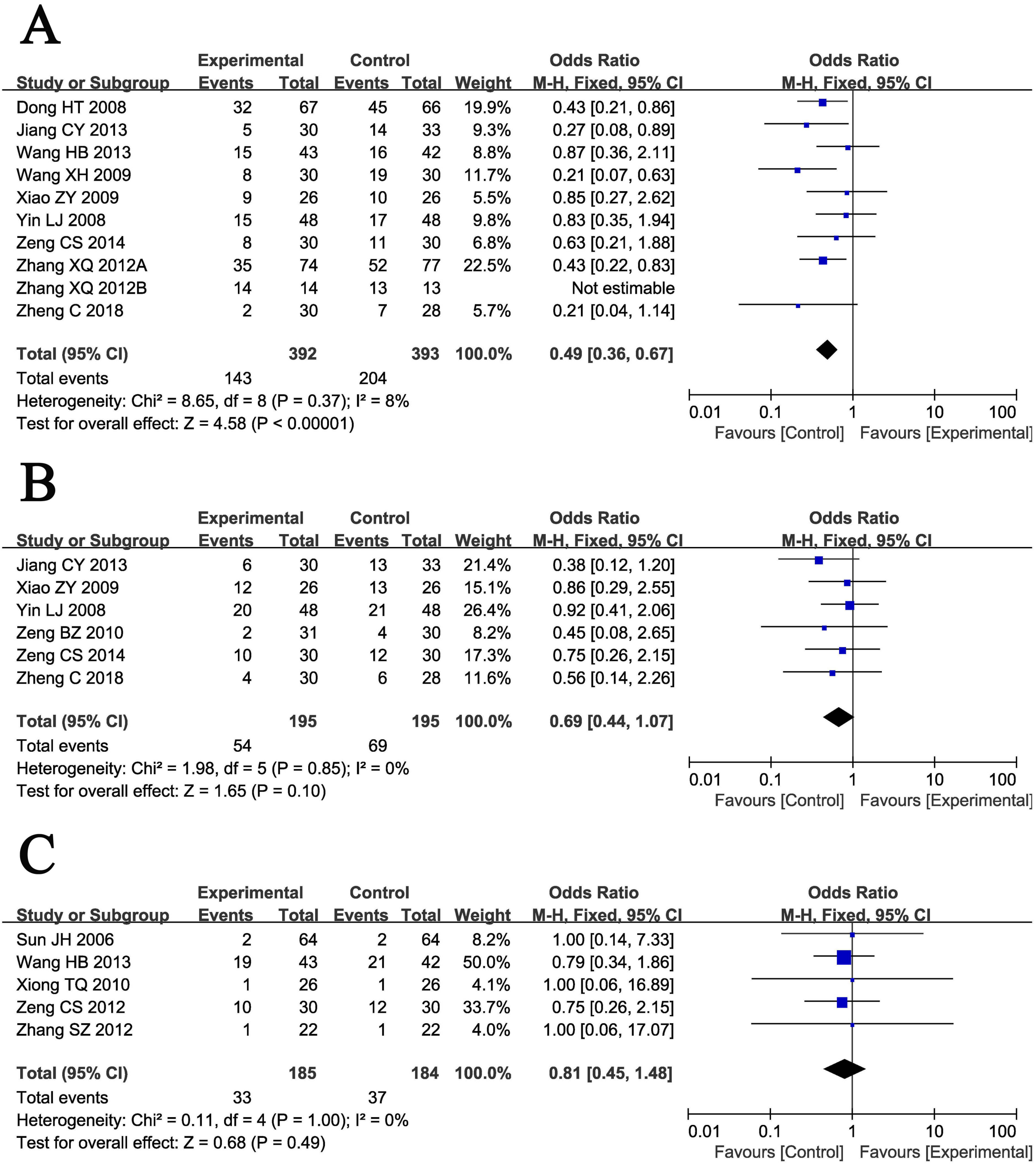
A
Experimental | Control | Odds Ratio | Odds Ratio
Study or Subgroup | Events | Total | Events | Total | Weight | M-H, Fixed, 95% CI | M-H, Fixed, 95% CI
Dong HT 2008 | 32 | 67 | 45 | 66 | 19.9% | 0.43 [0.21, 0.86]
Jiang CY 2013 | 5 | 30 | 14 | 33 | 9.3% | 0.27 [0.08, 0.89]
Wang HB 2013 | 15 | 43 | 16 | 42 | 8.8% | 0.87 [0.36, 2.11]
Wang XH 2009 | 8 | 30 | 19 | 30 | 11.7% | 0.21 [0.07, 0.63]
Xiao ZY 2009 | 9 | 26 | 10 | 26 | 5.5% | 0.85 [0.27, 2.62]
Yin LJ 2008 | 15 | 48 | 17 | 48 | 9.8% | 0.83 [0.35, 1.94]
Zeng CS 2014 | 8 | 30 | 11 | 30 | 6.8% | 0.63 [0.21, 1.88]
Zhang XQ 2012A | 35 | 74 | 52 | 77 | 22.5% | 0.43 [0.22, 0.83]
Zhang XQ 2012B | 14 | 14 | 13 | 13 | | Not estimable
Zheng C 2018 | 2 | 30 | 7 | 28 | 5.7% | 0.21 [0.04, 1.14]
Total (95% CI) | 392 | 393 | 100.0% | 0.49 [0.36, 0.67]
Total events | 143 | 204
Heterogeneity: Chi² = 8.65, df = 8 (P = 0.37); I² = 8%
Test for overall effect: Z = 4.58 (P < 0.00001)
0.01 0.1 1 10 100
Favours [Control] Favours [Experimental]
B
Experimental | Control | Odds Ratio | Odds Ratio
Study or Subgroup | Events | Total | Events | Total | Weight | M-H, Fixed, 95% CI | M-H, Fixed, 95% CI
Jiang CY 2013 | 6 | 30 | 13 | 33 | 21.4% | 0.38 [0.12, 1.20]
Xiao ZY 2009 | 12 | 26 | 13 | 26 | 15.1% | 0.86 [0.29, 2.55]
Yin LJ 2008 | 20 | 48 | 21 | 48 | 26.4% | 0.92 [0.41, 2.06]
Zeng BZ 2010 | 2 | 31 | 4 | 30 | 8.2% | 0.45 [0.08, 2.65]
Zeng CS 2014 | 10 | 30 | 12 | 30 | 17.3% | 0.75 [0.26, 2.15]
Zheng C 2018 | 4 | 30 | 6 | 28 | 11.6% | 0.56 [0.14, 2.26]
Total (95% CI) | 195 | 195 | 100.0% | 0.69 [0.44, 1.07]
Total events | 54 | 69
Heterogeneity: Chi² = 1.98, df = 5 (P = 0.85); I² = 0%
Test for overall effect: Z = 1.65 (P = 0.10)
0.01 0.1 1 10 100
Favours [Control] Favours [Experimental]
C
Experimental | Control | Odds Ratio | Odds Ratio
Study or Subgroup | Events | Total | Events | Total | Weight | M-H, Fixed, 95% CI | M-H, Fixed, 95% CI
Sun JH 2006 | 2 | 64 | 2 | 64 | 8.2% | 1.00 [0.14, 7.33]
Wang HB 2013 | 19 | 43 | 21 | 42 | 50.0% | 0.79 [0.34, 1.86]
Xiong TQ 2010 | 1 | 26 | 1 | 26 | 4.1% | 1.00 [0.06, 16.89]
Zeng CS 2012 | 10 | 30 | 12 | 30 | 33.7% | 0.75 [0.26, 2.15]
Zhang SZ 2012 | 1 | 22 | 1 | 22 | 4.0% | 1.00 [0.06, 17.07]
Total (95% CI) | 185 | 184 | 100.0% | 0.81 [0.45, 1.48]
Total events | 33 | 37
Heterogeneity: Chi² = 0.11, df = 4 (P = 1.00); I² = 0%
Test for overall effect: Z = 0.68 (P = 0.49)
0.01 0.1 1 10 100
Favours [Control] Favours [Experimental]

D

Study or Subgroup	Experimental Events	Experimental Total	Control Events	Control Total	Weight	Odds Ratio M-H, Fixed, 95% CI
Sun JH 2006	5	64	5	64	50.2%	1.00 [0.27, 3.64]
Xiong TQ 2010	2	26	2	26	20.1%	1.00 [0.13, 7.69]
Zhang SZ 2012	2	22	3	22	29.7%	0.63 [0.10, 4.22]
**Total (95% CI)**		**112**		**112**	**100.0%**	**0.89 [0.35, 2.29]**
Total events	9		10			

Heterogeneity: Chi² = 0.17, df = 2 (P = 0.92); I² = 0%
Test for overall effect: Z = 0.24 (P = 0.81)

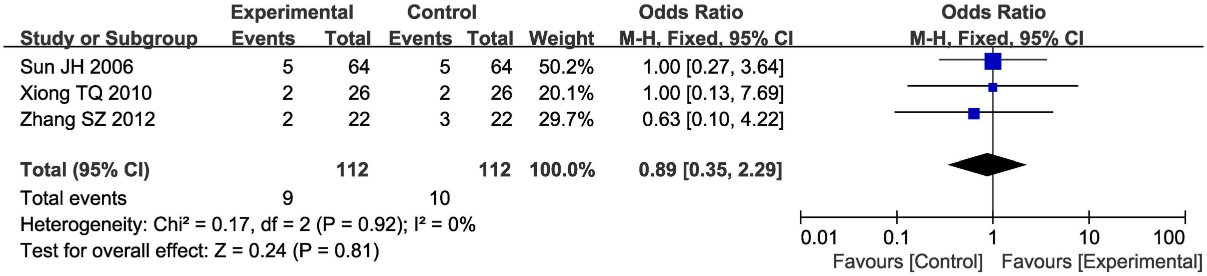

E

Study or Subgroup	Experimental Events	Experimental Total	Control Events	Control Total	Weight	Odds Ratio M-H, Fixed, 95% CI
Wang HB 2013	6	43	7	42	68.5%	0.81 [0.25, 2.65]
Zeng CS 2014	2	30	3	30	31.5%	0.64 [0.10, 4.15]
**Total (95% CI)**		**73**		**72**	**100.0%**	**0.76 [0.28, 2.06]**
Total events	8		10			

Heterogeneity: Chi² = 0.04, df = 1 (P = 0.84); I² = 0%
Test for overall effect: Z = 0.54 (P = 0.59)

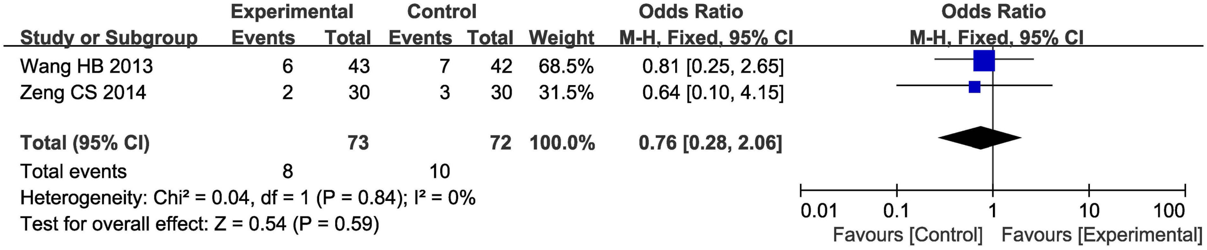

F

Study or Subgroup	Experimental Events	Experimental Total	Control Events	Control Total	Weight	Odds Ratio M-H, Fixed, 95% CI
Jiang CY 2013	11	30	24	33	75.7%	0.22 [0.07, 0.63]
Zheng C 2018	3	30	5	28	24.3%	0.51 [0.11, 2.37]
**Total (95% CI)**		**60**		**61**	**100.0%**	**0.29 [0.12, 0.69]**
Total events	14		29			

Heterogeneity: Chi² = 0.81, df = 1 (P = 0.37); I² = 0%
Test for overall effect: Z = 2.79 (P = 0.005)

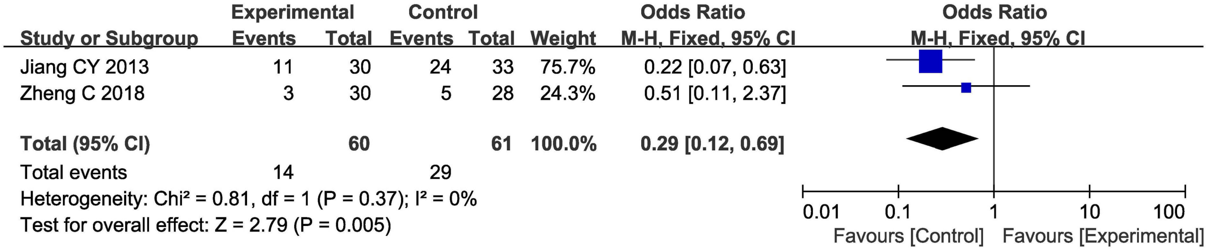

G

Study or Subgroup	Experimental Events	Experimental Total	Control Events	Control Total	Weight	Odds Ratio M-H, Fixed, 95% CI
Xie YF 2003	6	31	15	31	49.0%	0.26 [0.08, 0.80]
Zeng BZ 2010	7	31	16	30	51.0%	0.26 [0.08, 0.77]
**Total (95% CI)**		**62**		**61**	**100.0%**	**0.26 [0.12, 0.56]**
Total events	13		31			

Heterogeneity: Chi² = 0.00, df = 1 (P = 1.00); I² = 0%
Test for overall effect: Z = 3.37 (P = 0.0007)

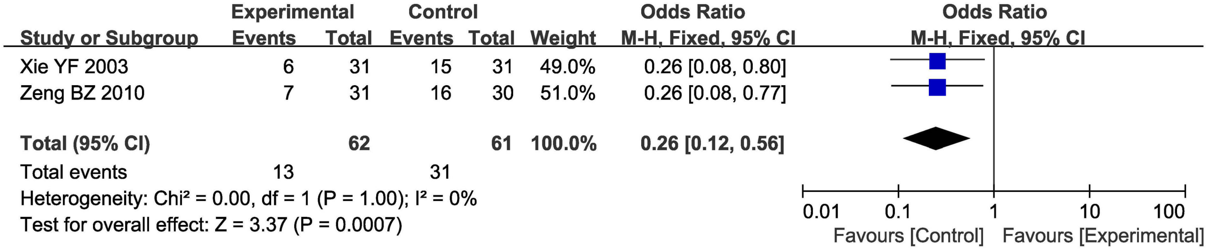

H

Study or Subgroup	Experimental Events	Experimental Total	Control Events	Control Total	Weight	Odds Ratio M-H, Fixed, 95% CI
Wang HB 2013	4	43	5	42	35.3%	0.76 [0.19, 3.05]
Zeng CS 2014	9	30	12	30	64.7%	0.64 [0.22, 1.87]
**Total (95% CI)**		**73**		**72**	**100.0%**	**0.68 [0.29, 1.60]**
Total events	13		17			

Heterogeneity: Chi² = 0.03, df = 1 (P = 0.85); I² = 0%
Test for overall effect: Z = 0.88 (P = 0.38)

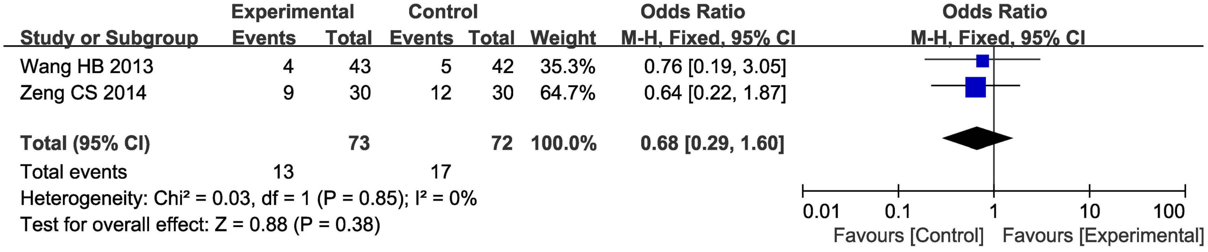

Supplementary Figure 2. Forest plot of the comparison of adverse effects including leukopenia (A), gastrointestinal adverse effects (B), nausea and vomiting (C), anorexia (D), thrombocytopenia (E), hepatotoxicity (F), myelosuppression (G) and anemia (H) between the experimental and control group. Control group, conventional treatment alone group; Experimental group, conventional treatment and JLC combined group; JLC, Jinlong capsule. The fixed-effects meta-analysis model (Mantel–Haenszel method) was used.

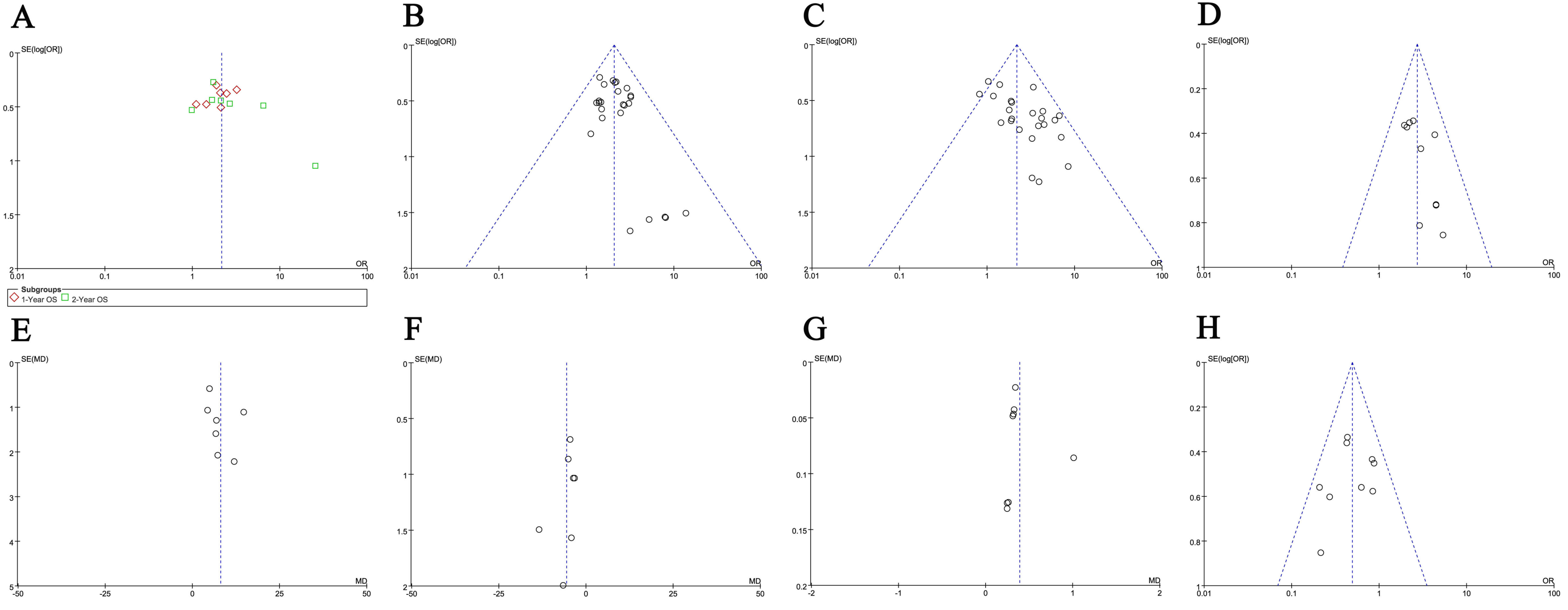
A
SE(log[OR])
OR
Subgroups
1-Year OS
2-Year OS
B
SE(log[OR])
OR
C
SE(log[OR])
OR
D
SE(log[OR])
OR
E
SE(MD)
MD
F
SE(MD)
MD
G
SE(MD)
MD
H
SE(log[OR])
OR

Supplementary Figure 3. Funnel plot of 12- and 24-month overall survival (OS, A), overall response rate (ORR, B), disease control rate (DCR, C), quality of life improved rate (QIR, D), $CD4^+$ (E), $CD8^+$ (F), $CD4^+/CD8^+$ (G), and leukopenia (H).

Supplementary Table 1. Quality assessment of non-randomized comparative studies

Study	Non-randomized studies								Additional criteria in comparative study				Total
	A	B	C	D	E	F	G	H	I	J	K	L	
Jia CH 2008[19]	2	1	2	1	2	1	2	0	2	2	2	2	19
Li H 2007[23]	2	1	2	1	1	2	2	0	2	2	2	2	19
Liang TJ 2005[24]	2	1	2	1	2	2	2	0	2	2	2	2	20
Liu ZY 2015[25]	2	1	2	1	2	1	2	0	2	2	2	2	19
Sun JH 2006[27]	2	1	2	2	2	2	2	0	2	2	2	2	21
Wang HB 2013[29]	2	1	2	2	2	2	2	0	2	2	2	2	21
Xiong TQ 2010[34]	2	1	2	2	2	1	2	0	2	2	2	2	20
Zhang XQ 2012[43]	2	1	2	2	2	2	2	0	2	2	2	2	21

Notes: The items are scored 0 (not reported), 1 (reported but inadequate) and 2 (reported and adequate).

A: A clearly stated aim; B: Inclusion of consecutive patients; C: Prospective collection of data; D: Endpoints appropriate to the aim of the study; E: Unbiased assessment of the study endpoint; F: Follow-up period appropriate to the aim of the study; G: Loss to follow up less than 5%; H: Prospective calculation of the study size; I: An adequate control group; J: Contemporary groups; K: Baseline equivalence of groups; L: Adequate statistical analyses.

Supplementary Table 2. Publication bias on therapeutic efficacy indexes (OS, ORR, DCR and QIR), immune function indexes ($CD4^+$, $CD8^+$, and $CD4^+/CD8^+$) and leucopenia.

Publication Bias	Therapeutic efficacy indexes					Immune function indexes			Adverse events
	12-month OS	24-month OS	ORR	DCR	QIR	$CD4^+$	$CD8^+$	$CD4^+/CD8^+$	Leucopenia
**Begg**	0.764	0.133	0.168	0.035	0.107	0.133	0.548	0.902	0.675
**Egger**	0.301	0.161	0.049	0.001	0.036	0.321	0.331	0.603	0.542
**Trim and fill analysis**									
before			$P < .0001$	$P < .0001$	$P < .0001$				
after			$P < .0001$	$P < .0001$	$P < .0001$				

Notes: Parameters discussed in over 7 papers were conducted bias analyses.

Abbreviations: OS: overall survival; ORR: overall response rate; DCR: disease control rate; QIR: quality of life improved rate.

Supplementary Table 3. Subgroup analyses of ORR and DCR between the experimental and control group.

Parameter	Factors at study level	Patients Con/Exp	Analysis method	Heterogeneity I^2 (%)	Heterogeneity *P*-value	Odds Ratio	95% CI	*P*-value
ORR	**Therapeutic regimen**							
	JLC+TACE	739/719	Fixed	0	0.96	1.93	1.56-2.38	<0.00001
	JLC+SST	157/157	Fixed	0	0.97	7.37	1.91-28.36	0.004
	JLC+RT	117/116	Fixed	0	0.93	3.02	1.72-5.31	0.0001
	**Study sample size**							
	>80	719/683	Fixed	0	0.83	2.06	1.64-2.59	<0.00001
	<80	379/380	Fixed	0	0.89	2.06	1.48-2.88	<0.0001
	**Type of control trials**							
	RCTs	677/654	Fixed	0	0.93	2.06	1.63-2.61	<0.00001
	non-RCTs	421/409	Fixed	0	0.69	2.07	1.52-2.82	<0.00001
DCR	**Therapeutic regimen**							
	JLC+TACE	672/653	Fixed	6	0.39	1.66	1.26-2.18	0.0003
	JLC+SST	157/157	Fixed	0	0.65	3.90	2.42-6.29	<0.00001
	JLC+RT	117/116	Fixed	0	0.76	4.50	1.46-13.86	0.009
	**Study sample size**							
	>80	652/617	Fixed	24	0.23	1.62	1.22-2.15	0.0009
	<80	379/380	Fixed	0	0.91	3.46	2.40-4.99	<0.00001
	**Type of control trials**							
	RCTs	610/588	Fixed	0	0.83	2.48	1.84-3.33	<0.00001
	non-RCTs	421/409	Random	63	0.01	2.09	1.11-3.94	0.02

Notes: Con, Control group (Conventional treatment alone group); Exp, Experimental group (Conventional treatment and JLC combined group).

Abbreviations: ORR: overall response rate; DCR: disease control rate; JLC: Jinlong capsule; TACE: transcatheter arterial chemoembolization; SST: Support and symptomatic treatment; RT: Radiotherapy; RCTs; randomized controlled trials.
